# Enamel surface evaluation after bracket debonding and different resin removal methods

**DOI:** 10.1590/2176-9451.20.2.061-067.oar

**Published:** 2015

**Authors:** Michele Machado Vidor, Rafael Perdomo Felix, Ernani Menezes Marchioro, Luciane Hahn

**Affiliations:** 1 Masters student in Clinical Dentistry, Radiology, Universidade Federal do Rio Grande do Sul (UFRGS), Santa Cecília, Rio Grande do Sul, Brazil; 2 Masters student in Dental Prosthesis, Pontifícia Universidade Católica do Rio Grande do Sul (PUCRS), Porto Alegre, Rio Grande do Sul, Brazil; 3 Adjunct professor, Pontifícia Universidade Católica do Rio Grande do Sul (PUCRS), Department of Dentistry, Porto Alegre, Rio Grande do Sul, Brazil; 4 Professor, São Leopoldo Mandic, School of Dentistry, Postgraduate program in Orthodontics, Porto Alegre, Rio Grande do Sul, Brazil

**Keywords:** Dental enamel, Dental debonding, Dental polishing

## Abstract

**OBJECTIVE:**

To assess enamel surface under scanning electron microscopy (SEM) after resin removal and enamel polishing procedures following brackets debonding, as well as compare the time required for these procedures.

**METHODS:**

A total of 180 deciduous bovine incisors were used. The enamel surface of each tooth was prepared and brackets were bonded with light cured Transbond XT composite resin. Brackets were removed in a testing machine. The samples were randomized and equally distributed into nine groups according to the resin removal and polishing technique: Group 1, 30-blade tungsten carbide bur in high speed; Group 2, 30-blade tungsten carbide bur in high speed followed by a sequence of 4 Sof-lex polishing discs (3M); Group 3, 30-blade tungsten carbide bur in high speed followed by Enhance tips (Dentsply). All groups were subdivided into (a) unpolished; (b) polished with aluminum oxide paste; and (c) polished with water slurry of fine pumice. Subsequently, the enamel surface was assessed and statistical analysis was carried out.

**RESULTS:**

There were statistically significant differences in enamel roughness and removal time among all groups. Groups 3a, 3b and 3c appeared to be the most efficient methods of removing resin with low damages to enamel. Groups 2a, 2b and 2c were the most time consuming procedures, and Group 2a caused more damages to enamel.

**CONCLUSION:**

The suggested protocol for resin removal is the 30-blade tungsten carbide bur in high speed followed by Enhance tips and polishing with aluminum oxide paste. This procedure seems to produce less damages and is less time consuming.

## Abstract (pt)

**OBJETIVO:**

avaliar a superfície do esmalte, por meio de microscopia eletrônica de varredura, após descolagem de braquetes e o polimento do esmalte, com diferentes técnicas de remoção de resina, bem como comparar o tempo necessário para a realização do procedimento.

**MÉTODOS:**

foram utilizados 180 dentes bovinos decíduos. A superfície do esmalte dos dentes foi preparada e realizada a colagem dos braquetes com resina Transbond XT. Os braquetes foram removidos em máquina de ensaio mecânico. Foram formados, aleatoriamente, nove grupos, de acordo com o tipo de remoção da resina e de polimento, sendo: Grupo 1, broca de tungstênio de 30 lâminas em alta rotação; Grupo 2, broca de tungstênio de 30 lâminas em alta rotação e sequência de quatro discos Soflex (3M); Grupo 3, broca de tungstênio de 30 lâminas em alta rotação e pontas de acabamento Enhance (Dentsply). Todos os grupos foram subdividido em (a) sem polimento, (b) polimento com pasta de óxido de alumínio e (c) polimento com pedra-pomes. As superfícies foram avaliadas e foi realizada análise estatística.

**RESULTADOS:**

houve diferença estatística entre os grupos em relação à rugosidade de superfície e ao tempo de remoção. Os grupos 3a, 3b e 3c demonstraram ser os métodos mais eficientes de remoção da resina, promovendo pouco dano à superfície do esmalte. Os grupos 2a, 2b e 2c consumiram maior tempo de procedimento, e o grupo 2a causou maior dano ao esmalte.

**CONCLUSÃO:**

sugere-se como protocolo de remoção o uso de broca de tungstênio de 30 lâminas para remoção do maior volume da resina, pontas de acabamento Enhance e polimento com a pasta de óxido de alumínio, por produzirem menor dano.

## INTRODUCTION

In Orthodontics, as in other dental specialties, there is an ongoing urge to simplify technical procedures in order to achieve the goals with quality and minimal discomfort.^1^ Acid etching of tooth surface, introduced by Buonocore^2^ in 1955, is an example and represents a major breakthrough in Dentistry. The adhesive technique allowed bracket direct bonding with significant reduction of bands placement around teeth, resulting in faster, easier and more accurate accessories positioning, also making the procedure more comfortable to patients.^1^ Advances in the technology of bonding material allowed this procedure to become safe and efficient due to its good mechanical and physical properties. However, bracket removal and enamel surface polishing after debonding have become a concern. The search for a safe and efficient method attracted the attention of many researchers, which resulted in the introduction of numerous tools and techniques.^2^^-^ 21 Nevertheless, the techniques that provide efficient enamel surface polishing present a wide clinical sequence. Thus, many clinicians create their own methods of resin removal and enamel polishing based on trial and error without knowing the actual damage they may be causing to patient's enamel.^3 ^Therefore, no consensus has been reached regarding the best resin removal technique promoting less damage to enamel surface.^4^^,^ 5

Thus, the main purpose of this study is to assess the enamel surface after different resin removal and enamel polishing techniques after bracket debonding. Assessment was carried out by means of scanning electron microscopy (SEM). Moreover, the present study also aimed to compare the time required for these procedures, and present a simplified and efficient protocol aiming at lower loss and damage to the enamel.

## MATERIAL AND METHODS

A total of 180 bovine deciduous incisors were used in this study. In selecting the sample, the following inclusion criteria were applied: integrity of tooth enamel, no caries, fractures or cracks visible to the naked eye. For preparation of specimens, the teeth were sectioned at the tooth cervix, and only the dental crowns were used. The remaining dental pulp in the crown was removed using a dental probe. Subsequently, the crowns were placed on wax, with the buccal surface against a glass plate so as to allow most part of the flat surface of enamel to stay parallel to the ground and perpendicular to the sidewalls of the PVC ring. In this position, the crowns were fixed by heating the wax around the teeth with a heated wax scraper. Afterwards, standard PVC rings, with 20 mm of internal diameter and height, were positioned in such a way so as to involve the entire crown. Self-curing acrylic resin was then poured on them. Once the setting time of acrylic resin had passed, the samples were washed with water vapor pressure in order to remove all the wax. Samples were then stored under immersion in distilled water, at room temperature, in a sealed plastic container until bracket bonding.

The bracket bonding area was determined clinically and by inspection on the flat portion of the buccal surface of the dental crown and closest to its center. Enamel surfaces were prepared for bonding as described below:

1) Prophylaxis with rubber cup in low rotation, using pumice and water for 10 seconds.
2) Washing with distilled water for 10 seconds.
3) Drying with compressed air, free from oil and water, for 10 seconds at a distance of 5 cm.
4) Acid etching with 37% phosphoric acid for 15 seconds, subsequently washed with distilled water for 10 seconds and dried with compressed air for 10 seconds.
5) Adhesive application on etched enamel (Transbond XT).
6) Application of composite resin (Transbond XT) on the bracket basis and positioning on the tooth with a bracket placing forceps with enough manual pressure for the disposal of excess material removed with a dental probe.
7) Light curing of adhesive and composite resin.

A stainless steel lower incisor bracket was bonded by one single operator on each one of the 180 teeth used in the sample. Bracket base surface was 10.47 mm^2^, as measured by a digital caliper. After the bonding procedure, the samples were immersed in distilled water and stored in a closed container, in an incubator, set at 37° C for 24 hours. Accessories removal was accomplished through a mechanical testing machine, operated at 0,5 mm/min, in which a round section stainless steel wire (0.018-in) was positioned holding the bracket wings while keeping it parallel to the direction of the force. After debonding, the samples were assessed using a stereoscopic microscope (10x magnification) operated by a single calibrated investigator so as to evaluate adhesive remnant index (ARI) according to the classification criteria established by Årtun and Bergland^6^ (Table 1). Excess resin surrounding the bracket base was not considered.

**Table 1 -:** Adhesive remnant index idealized by Årtun and Bergland.6

Score 0	No adhesive left on the tooth enamel
Score 1	Less than half adhesive left on the tooth enamel
Score 2	More then half adhesive left on the tooth enamel
Score 3	All adhesive left on the tooth enamel with a distinct impression of the bracket mesh

The samples were randomly divided into nine groups (n = 20) according to the resin removal technique and the polishing procedure executed or not at the enamel surface (Table 2).

**Table 2 -:** Division of groups regarding resin removal and polishing techniques.

	Unpolished	Polished with aluminium oxide	Polished with pumice
Tungsten drill – 30 blades	Group 1a	Group 1b	Group 1c
Tungsten drill – 30 blades + sequence of four Sof-lex disc–discs	Group 2a	Group 2b	Group 2c
Tungsten drill – 30 blades + Enhance finishing tips	Group 3a	Group 3b	Group 3c

Resin removal in Groups 1a, 1b and 1c was carried out only with tungsten carbide drill (30 blades, 9714FFJET) in high speed, without irrigation and light force application moving in one direction. In Groups 2a, 2b and 2c, the same technique was used with the tungsten carbide drill for removal of the largest volume of resin, followed by the sequence of four Sof-lex (3M) discs in low rotation. In Groups 3a, 3b and 3c, after the use of a tungsten carbide drill, resin removal was performed with Enhance finishing tips (Dentsply). Tungsten carbide burs, Sof-lex (3M) discs and Enhance finishing tips were replaced every five samples. Resin removal was deemed complete when the surface seemed to be smooth and without resin by the naked eye under illumination of the light reflector. The time for complete removal was registered in seconds. After the removal procedure, enamel surface polishing of Groups 1b, 2b and 3b was carried out with aluminum oxide paste (Enamelize Cosmedent) and felt disc (Flexibuff Cosmedent); while in Groups 1c, 2c and 3c polishing was performed with pumice and a rubber cup. In Groups 1a, 2a and 3a, no surface polishing was performed.

Specimens were examined by scanning electron microscopy (Philips XL 30), under magnification of 500 and 1500 x. Images were printed for evaluation of enamel surface by a single, previously calibrated investigator. Assessment was based on the surface roughness index (SRI) according to the classification criteria shown in Table 3.

**Table 3 -:** Surface roughness index.

Score 1	Acceptable surface with thin and scattered grooves
Score 2	Slightly rough surface, with some thin and other thicker grooves
Score 3	Rough surface, several thick grooves over the entire tooth surface
Score 4	Very rough surface, deep and thick grooves over the entire surface

Source: Closs, Reston and Falster.7

The non-parametric Kruskal-Wallis test was employed to assess adhesive remnant index (ARI) and surface roughness index (SRI). In order to compare resin removal time, factorial analysis of variance test was carried out. This test compares a study variable (time) considering two factors (polishing and group) in order to verify whether the polishing-group interaction is significant. In other words, whether or not these two factors combined interfere in the variable. The groups were compared by means of analysis of variance (ANOVA) and Tukey's multiple comparison tests.

## RESULTS AND DISCUSSION

The present study aimed to assess the enamel surface resulting from different resin removal techniques after bracket debonding, followed or not by polishing. Assessment was conducted to determine which technique causes less damage to enamel surface and provides better clinical time for execution. Despite the more frequent use of human premolars in adhesion tests in Orthodontics,^8^ the use of bovine teeth became a viable alternative due to difficulty obtaining human teeth for *in vitro* studies.^9^ Even though bovine deciduous teeth offer lower bond strength, researches have concluded that they can be used as substitutes in laboratory studies, since they present a fairly regular surface and similarities in composition and mechanical properties to human teeth.^10^ In view of difficulties obtaining human teeth and scientific support concerning the use of bovine teeth, we opted to use the latter for the present study. 

After bracket removal, the teeth surface were assessed as to the amount of remaining resin, according to the classification criteria of Årtun and Bergland^6^ for ARI. The non-parametric Kruskal-Wallis test revealed that there was no significant difference for the adhesive remnant index (ARI) among the nine groups compared (P = 0.395) (Table 4), with predominance of scores 2 and 3. These results indicate that more than half of resin was left on the tooth (score 2) or all resin was left on the tooth with the bracket mash impression (score 3). Since there were no statistically significant differences regarding the amount of resin remaining after bracket debonding, it was possible to compare resin removal time and enamel polishing.

**Table 4 -:** Comparison among adhesive remnant index scores among the nine groups.

Group	Adhesive remnant index							
	Score 0		Score 1		Score 2		Score 3	
	n	%	n	%	n	%	n	%
1a	-	-	-	-	5	25.0	15	75.0
1b	-	-	-	-	2	10.0	18	90.0
1c	-	-	-	-	4	20.0	16	80.0
2a	-	-	-	-	2	10.0	18	90.0
2b	-	-	-	-	1	5.0	19	95.0
2c	-	-	-	-	1	5.0	19	95.0
3a	-	-	-	-	5	25.0	15	75.0
3b	-	-	-	-	2	10.0	18	90.0
3c	-	-	-	-	4	20.0	16	80.0
Total	-	-	-	-	26	14.4	154	85.6

The results obtained with regard to resin removal and enamel surface polishing time by means of factorial analysis of variance showed that the effect of group / type of polishing procedure interaction was not significant; however, the factors polishing and group, when analyzed separately, revealed a statistically significance difference (Table 5).

**Table 5 -:** Results of the factorial analysis of variance when comparing time considering two factors: group and type of polishing procedure.

Polishing procedure	Number of cases	Mean ± SD	F	P
Tungsten drill				
Unpolished	20	46.95^A ^± 17.58	6.35	0. 003
Polished with aluminium oxide	20	67.85^B ^± 20.41		
Polished with pumice	20	60.00^B ^± 18.11		
Tungsten drill + Sof-lex disc				
Unpolished	20	79.30^A ^± 19.32	8.56	0. 001
Polished with aluminium oxide	20	105.95^B ^± 20.98		
Polished with pumice	20	91.10^AB ^± 20.89		
Tungsten drill + Enhance finishing tips				
Unpolished	20	34.25^A ^± 14.07	9.64	0. 000
Polished with aluminium oxide	20	52.80^B ^±12.03		
Polished with pumice	20	45.10^B ^± 14.07		

By means of this test, comparisons were made between groups and between the types of polishing procedures in relation to the variable time. Analysis of variance (ANOVA) and Tukey's multiple comparison test results revealed a statistically significant difference between the types of polishing procedure for all groups, also showing that the time spent in the unpolished samples is significantly lower than that spent in the samples polished with aluminum oxide and pumice, which did not differ among each other (Table 6).

**Table 6 -:** Comparison of time (seconds) between the types of polishing procedure for each group.

Sources of variation	Sum of squares	DF	Medium square	F	P
Group	73166.8	2	36583.4	115.75	0.000
Type of polishing procedure	14595.2	2	7297.6	23.09	0.000
Group x type of polishing procedure	470.3	4	117.5	0.37	0.828
Error	54045.2	171	316.1		
Total	898364	180			

* SD = Standard deviation.

** Means followed by the same letter do not differ.

There were significant differences between all groups in terms of resin removal time. The tungsten drill + Sof-lex discs groups featured significantly superior time in comparison to the other groups, associated or not with enamel polishing. The tungsten drill + Enhance finishing tips groups presented the smallest resin removal time among the polished groups. The time for tungsten drill + Enhance finishing tips unpolished group did not significantly differ from procedures involving tungsten drill, only (Table 7). Our findings regarding the longer resin removal time associated with the use of Sof-lex disc corroborate those found in the literature.^9^

**Table 7 -:** Comparison of time (seconds) among groups for each type of polishing procedure.

Group	Number of cases	Mean ± SD	F	P
Unpolished				
Tungsten drill	20	46.95^A ^± 17.58	36.78	0.000
Tungsten drill + Sof-lex disc	20	79.30^B ^± 19.32		
Tungsten drill + Enhance finishing tips	20	34.2^A ^± 14.07		
Polished with aluminium oxide				
Tungsten drill	20	67.85^A ^± 20.41	44.96	0.000
Tungsten drill + Sof-lex disc	20	105.95^B ^± 20.98		
Tungsten drill + Enhance finishing tips	20	52.80^C ^± 12.03		
Polished with pumice				
Tungsten drill	20	60.00^A ^± 18.11	34.33	0.000
Tungsten drill + Sof-lex disc	20	91.10^B ^± 20.89		
Tungsten drill + Enhance finishing tips	20	45.10^C ^± 14.07		

* SD = Standard deviation.

** Means followed by the same letter do not differ.

**Figure 1 -:**
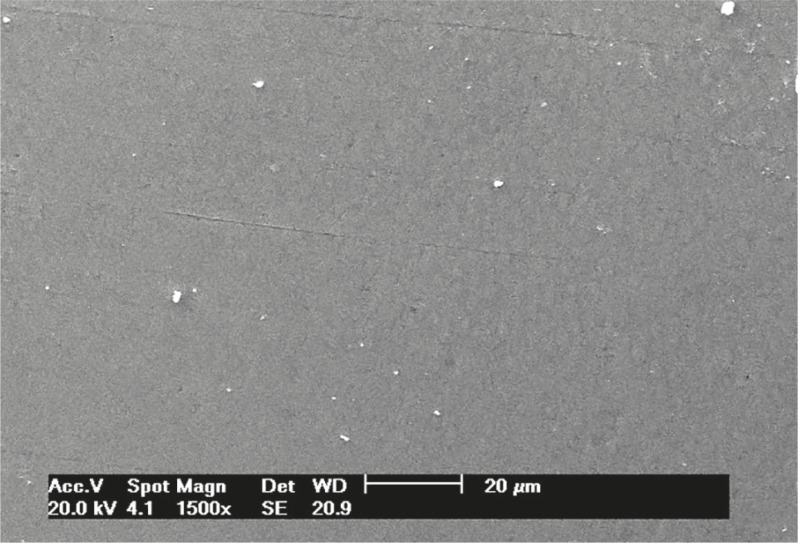
Micrography of healthy bovine tooth enamel surface-(control).

Scanning electron microscopy (SEM) allows better visualization of the enamel surface after different methods of resin removal and enamel polishing are carried out.^1^^,^ 11 ^,^ 13 ^,^ 16 ^,^ 17 ^,^ 18 ^,^ 23 In order to enable a comparative analysis of the different techniques employed, the enamel surface was assessed according to the surface roughness index (SRI).^19^ A healthy deciduous bovine tooth was used as control (Fig 1).

All methods effectively removed all adhesive remnant after debonding, and also produced grooves on the enamel surface that varied in depth, thereby corroborating several studies which have obtained the same results.^7^^,^ 12 ^,^ 14 ^,^ 22 ^,^ 24

The results of the Kruskal-Wallis non-parametric test for the surface roughness index demonstrated statistically significant differences among the nine groups compared (Table 8). In which it is observed:

**Table 8 -:** Scores of surface roughness index comparison among the nine groups.

Group	Surface roughness index							
	Score 1		Score 2		Score 3		Score 4	
	n	%	n	%	n	%	n	%
1a	3	15.0	12	60.0	5	25.0	-	-
1b	7	35.0	8	40.0	3	15.0	2	10.0
1c	4	20.0	11	55.0	5	25.0	-	-
2a	-	-	6	30.0	10	50.0	4	20.0
2b	2	10.0	9	45.0	9	45.0	-	-
2c	3	15.0	10	50.0	6	30.0	1	5.0
3a	10	50.0	8	40.0	2	10.0	-	-
3b	13	65.0	6	30.0	1	5.0	-	-
3c	10	50.0	7	35.0	2	10.0	1	5.0
Total	52	28.9	77	42.8	43	23.9	8	4.4

**Figure 2 -:**
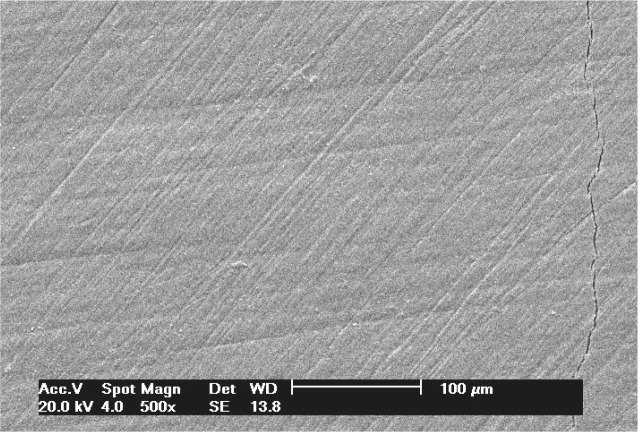
Micrography of enamel surface after resin removal with Tungsten drill + Sof-lex(r) discs without polishing (Group 2a).

**Figure 3 -:**
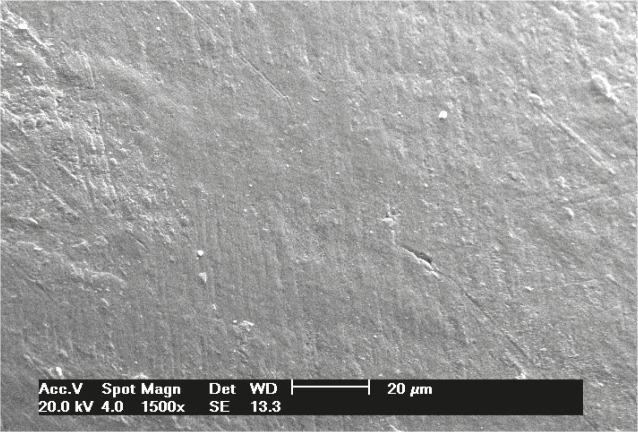
Micrography of enamel surface after resin removal with tungsten drill without polishing (Group 1a).

**Figure 4 -:**
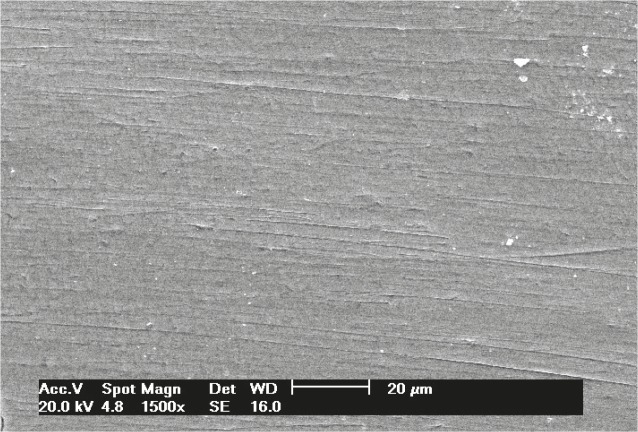
Micrography of enamel surface after resin removal with tungsten drill and polishing with aluminum oxide (Group 1b).

**Figure 5 -:**
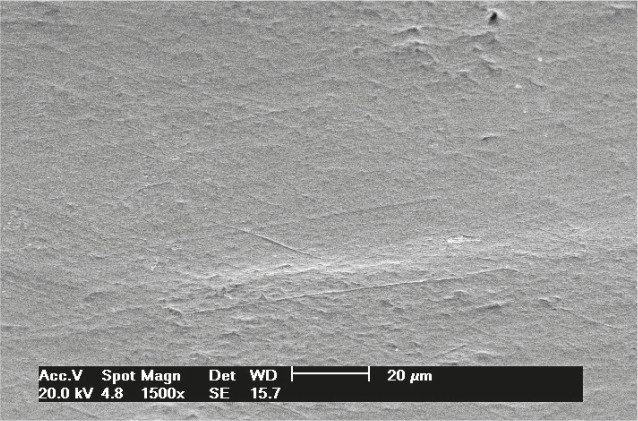
Micrography of enamel surface after resin removal with tungsten drill and polishing with pumice (Group 1c).

**Figure 6 -:**
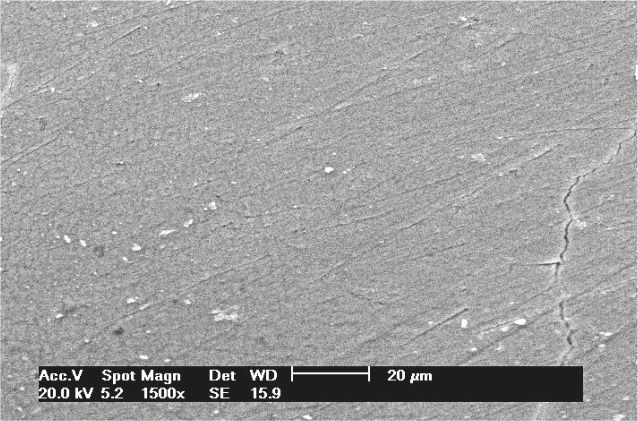
Micrography of enamel surface after resin removal with tungsten drill + Sof-lex discs and polishing with aluminium oxide (Group 2b).

**Figure 7 -:**
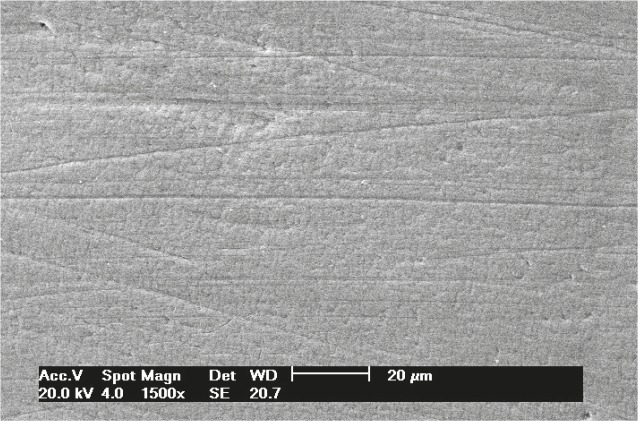
Micrography of enamel surface after resin removal with tungsten drill + Sof-lex discs and polishing with pumice (Group 2c).

**Figure 8 -:**
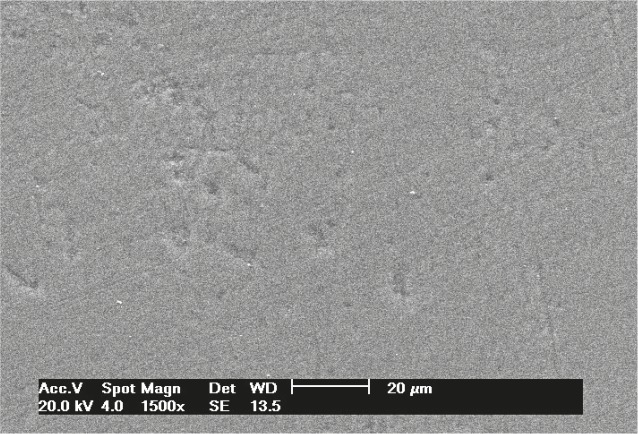
Micrography of enamel surface after resin removal with tungsten drill + Enhance finishing tips without polishing (Group 3a).

**Figure 9 -:**
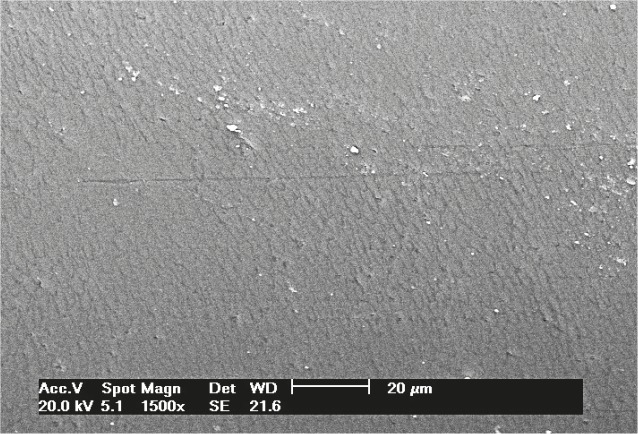
Micrography of enamel surface after resin removal with tungsten drill + Enhance finishing tips and polishing with aluminium oxide (Group 3b).

**Figure 10 -:**
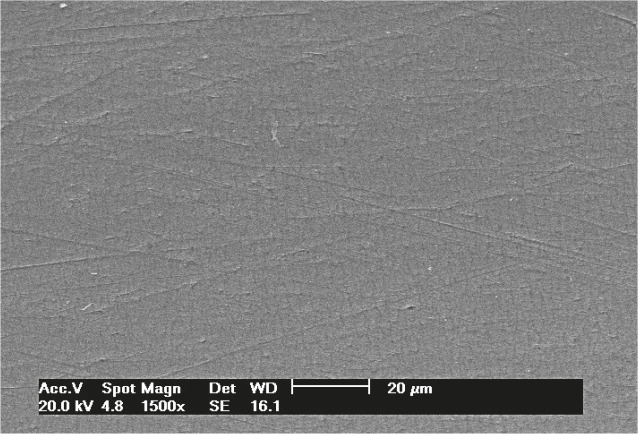
Micrography of enamel surface after resin removal with tungsten drill + Enhance finishing tips and polishing with pumice (Group 3c).

" Group 2a presented the highest scores (Fig 2);

" Groups 1a, 1b, 1c, 2b and 2c featured intermediate scores (Figs 3 to 7);

" Groups 3a, 3b and 3c featured the lowest scores (Figs 8, 9, 10).

Results showed that the group causing more damage to enamel surface was Group 2a (tungsten drill + Sof-lex discs without enamel polishing), and that the methods that provided an enamel surface with fewer grooves were those in which resin removal was performed with tungsten drill + Enhance finishing tips, followed or not by enamel surface polishing. According to Tüfekçi *et al*,^11^ remnant resin removal with Sof-lex discs produces deeper wear, beyond maximum average depth, causing more damage to the enamel. Opposed to these findings, Zarrinnia, Eid and Kehoe^18^ found best results when polishing the enamel surface with Sof-lex discs. Nevertheless, some researchers^18^ observed greater roughness when resin was removed with Enhance finishing tips; however, previous studies^17^ have suggested the use of these abrasive tips on resin removal protocol with a view to minimizing the grooves produced by drills and discs. These findings corroborate the results obtained in the present study in which finishing tips produced the lowest roughness scores, thereby microscopically showing better surface smoothness, similarly to healthy enamel.

Several authors emphasize the importance of enamel polishing after bracket debonding and resin removal based on improvements of enamel surface after this procedure.^1^^,^ 12 ^,^ 13 ^,^ 17 ^,^ 19 ^,^ 20 ^,^ 22 ^,^ 24 ^,^ 26

In addition, Fonseca et al^25 ^reported that polishing not only increases surface smoothness, but also provides a special shine and prevents plaque retention. Although we have not found statistically significant differences among groups, the literature shows the importance of final polishing. Additionally, even though there is no statistical difference regarding the best polishing technique, electron microscopy suggests smoother enamel surfaces when polishing is carried out with aluminum oxide paste in comparison to pumice stone. Visually, it also presented a glossy surface.

## CONCLUSION

Based on the results of this study it is reasonable to conclude that all techniques employed to remove remnant resin from enamel surface promoted grooves and, although no statistically significant difference was found with regard to polishing in the present study, we fully agree with the literature about the importance of enamel polishing. Additionally, even though there were no statistical differences concerning the best polishing technique, electron microscopy suggests smoother enamel surfaces when polishing is performed with aluminum oxide paste in comparison to pumice stone. Visually, it also presented a glossy surface. 

Among the aspects analyzed herein, the use of tungsten drill (30 blades), in unidirectional movements, is recommended to remove large volumes of resin remnant, followed by Enhance finishing tips with gentle pressure and polishing with aluminum oxide paste. This protocol promotes better enamel surface smoothness in addition to a reduced procedure time.
